# Aged mice display altered numbers and phenotype of basophils, and bone marrow-derived basophil activation, with a limited role for aging-associated microbiota

**DOI:** 10.1186/s12979-018-0135-6

**Published:** 2018-11-29

**Authors:** Adriaan A. van Beek, Floris Fransen, Ben Meijer, Paul de Vos, Edward F. Knol, Huub F. J. Savelkoul

**Affiliations:** 1 Cell Biology and Immunology Group, Wageningen University, Wageningen, the Netherlands; 2 Top Institute Food and Nutrition, Wageningen, the Netherlands; 3 Department of Immunology, Erasmus University Medical Center, Dr. Molewaterplein 40, 3015 GD Rotterdam, the Netherlands; 4 Pathology and Medical Biology, University of Groningen, Groningen, the Netherlands; 5 Department of Immunology, University Medical Center, Utrecht, the Netherlands; 6 Dermatology/Allergology, University Medical Center, Utrecht, the Netherlands

**Keywords:** Aging, Basophils, Immunity, Microbiota, Bone marrow, Spleen

## Abstract

**Background:**

The influence of age on basophils is poorly understood, as well as the effect of aging-associated microbiota on basophils. Therefore, we studied the influence of aging and aging-associated microbiota on basophil frequency and phenotype, and differentiation from basophil precursors.

**Results:**

Basophils became more abundant in bone marrow (BM) and spleens of 19-month-old mice compared with 4-month-old mice. Aged basophils tended to express less CD200R3 and more CD123, both in BM and spleen. Differences in microbiota composition with aging were confirmed by 16S sequencing. Microbiota transfers from young and old mice to germ-free recipients revealed that CD11b tended to be lowered on splenic basophils by aging-associated microbiota. Furthermore, abundance of *Alistipes*, *Oscillibacter*, *Bacteroidetes RC9 gut group*, and *S24–7* family positively correlated and CD123 expression, whereas *Akkermansia* abundance negatively correlated with basophils numbers.

Subsequently, we purified FcεRIα^+^CD11c^−^CD117^−^ BM-derived basophils and found that those from aged mice expressed lower levels of CD11b upon stimulation. Higher frequencies of IL-4^+^ basophils were generated from basophil precursors of aged mice, which could be reproduced in basophils derived from germ-free recipients of aging-associated microbiota.

**Conclusions:**

Collectively, these results show the influence of aging on basophils. Furthermore, this study shows that aging-associated microbiota altered activation of BM-derived basophils in a similar fashion as observed in BM-derived basophils from aged mice.

## Background

The human adult gut contains about 10^13^–10^14^ bacteria [1, 2], which is comparable to the number of human cells in the total body of a 30-year-old adult [3]. These commensal gut microbiota modulate the immune system [4] and contribute to immune homeostasis in the mucosal immune system [5]. Gut microbiota play an important modulatory role beyond mucosal immunity, for instance by changing the stem cell niche in the bone marrow (BM) [6]. Furthermore, absence of microbe-derived peptidoglycan in the circulation impairs the killing by BM neutrophils of *Salmonella pneumoniae* and *Staphylococcus aureus* [7]. In addition, in the absence of microbiota, CD123 (IL-3Rα) expression on basophil precursors was upregulated, thereby enhancing their responsiveness to interleukin (IL) 3 [8].

During aging the immune system develops several defects and undergoes various changes in differentiation, distribution, and activation [9]. Anti-parasitic immune responses in aged mice are impaired [10], which may indicate age-related changes in basophil function [11]. With aging, gut microbiota composition changes [12]. Basophil hematopoiesis and function are regulated by gut microbiota. Absence of gut microbiota lead to increased basophil frequencies and enhanced T helper (Th) 2 immune responses [8]. In addition, basophils express Toll-like receptor (TLR) 2 and TLR4, and respond to microbial ligands like peptidoglycan [13] and lipopolysaccharide (LPS) [14]. Histamine release and sensitivity of basophils from elderly were reported to be increased upon anti-immunoglobulin (Ig) E stimulation [15], but in a different study, no age-related difference was found in histamine release of human blood basophils upon anti-IgE or anti-IgG4 stimulation [16]. Basophil counts were not associated with frailty or mortality in elderly women [17, 18]. Basophil frequencies and absolute numbers decreased in blood from healthy elderly volunteers and patients suffering from Alzheimer’s disease [19, 20]. It is, however, largely unknown what effect age has on basophil differentiation and function.

Basophils are granulocytes which are involved in mounting and perpetuating Th2-mediated responses [21]. Basophils are an important source of IL-4 and IL-13, which direct the immune response towards Th2 type responses [22]. After IgD crosslinking, basophils produced IL-1, IL-4 and B cell activating factor (BAFF), supporting B cell functions [23]. Basophils are the major source of IL-4 after *Streptococcus pneumoniae* infection, contributing to humoral memory immune responses [24]. In addition, the basophil is crucial in the pathophysiology of systemic lupus erythematosus [25, 26], and its counts are a marker for disease activity [27]. Recently, basophil infiltration into tumors after depletion of regulatory T cells was implicated in tumor rejection via C-C motif chemokine ligand (CCL) 3- and CCL4-mediated recruitment of CD8^+^ T cells to tumors [28], indicating a role beyond classical Th2 responses.

Basophil differentiation and functions are dependent on IL-3 or thymic stromal lymphopoietin (TSLP) [29]. Basophils can be activated in an IgE-dependent and IgE-independent manner. Regarding IgE-dependent activation, FcεRIα crosslinking by complexes of IgE and antigen activates basophils, resulting in IL-4 and IL-13 production [30]. Basophils express IL-18R and IL-33R (ST2), and upon stimulation with IL-18 and IL-33, basophils produce IL-4, IL-6, IL-13, granulocyte-macrophage colony stimulating factor (GM-CSF), and several chemokines [31]. This effect is further enhanced in the presence of IL-3 [32]. CD200R3-mediated activation of basophils leads to IL-4 production in vitro, and to anaphylaxis in vivo [33].

Here we studied the influence of the aging-associated microbiota on basophil frequency and phenotype, and differentiation from precursors of basophils. We compared basophils from young germ-free recipients of microbiota of 4-month-old to young germ-free recipients of microbiota of 18-month-old mice. In addition, we studied changes in frequency and phenotype of basophils in BM and spleen, correlation between microbial genera and basophils, and changes in differentiation from precursors of basophils during aging by comparing 4-month-old and 18-month-old mice.

## Results

### Basophils become more abundant during aging and display a changed phenotype

**Fig. 1:**
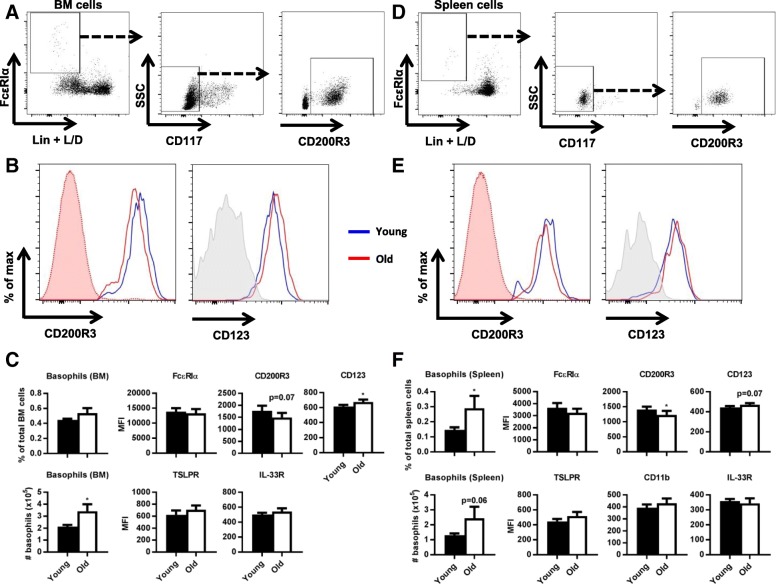
Effect of age on basophils in the bone marrow and the spleen. **a** Flow cytometric analysis of basophils in the BM, defined as live Lin^−^FcεRIα^+^CD117^−^CD200R3^+^. **b** Surface expression on BM basophils of CD200R3 and CD123. Representative example of a young (open blue) and an old mouse (open red). All BM cells from the same old (filled red) are shown in the CD200R3 plot. Isotype staining for CD123 is shown in grey. **c** Quantification of mean frequencies and absolute numbers of BM basophils or MFI on BM basophils of FcεRIα, CD200R3, IL-33R, and TSLPR. **d** Flow cytometric analysis of basophils in the spleen, defined as live Lin^−^FcεRIα^+^CD117^−^CD200R3^+^. **e** Surface expression on spleen basophils of CD200R3 and CD123. Representative example of a young (open blue) and an old mouse (open red). All spleen cells from the same old (filled red) are shown in the CD200R3 plot. Isotype staining for CD123 is shown in grey. **f** Quantification of mean frequencies and absolute numbers of spleen basophils or MFI on spleen basophils of FcεRIα, CD200R3, TSLPR, IL-33R, and CD11b. * = *p* < 0.05. Data represent *n* = 10 mice per group. BM = bone marrow; L/D = live/dead stain; Lin = lineage (CD3, CD4, CD8, CD11c, CD19, CD45R/B220, Ly6C/Ly6G (Gr-1), NK1.1, TER-119), with CD11b additionally in BM; MFI = median fluorescence intensity; SSC = side scatter

To identify the effect of age on basophil frequencies and phenotype, we analyzed frequencies of lineage (Lin)^−^CD117^−^FcεRIα^+^CD200R3^+^ basophils in mouse BM (Fig. 1a) and spleen (Fig. 1d), as well as absolute numbers. By comparing young and old mice, we found that the frequencies of basophils in the BM were similar (Fig. 1c), but were increased in the spleen of aged mice (*p* = 0.03; Fig. 1f), whereas absolute numbers were increased in BM (*p* = 0.02) and spleen (*p* = 0.06; Fig. 1c, f). The phenotype of basophils changed in both BM and spleen. CD200R3 expression tended to decrease on basophils in the BM (*p* < 0.08; Fig. 1b, c) and was decreased in the spleen of aged mice (*p* = 0.04; Fig. 1e, f), but CD123 expression was increased in aged basophils in the BM (p = 0.04) and tended to be increased in the spleen (*p* = 0.07). No age-related changes in FcεRIα, TSLPR, CD11b, and IL-33R (Fig. 1c, f) were observed.

### Microbiota composition changes with age and after microbiota transfer of young and aged mice

**Fig. 2:**
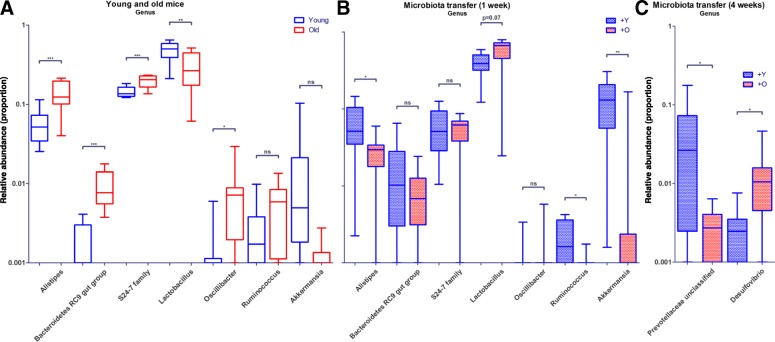
Effect of age and age-related microbiota on microbiota composition. **a** Bacterial genera that were significantly different in abundance in young or old mice. **b**-**c** Bacterial genera that were significantly different between germ-free recipients of young or old microbiota, after 1 week or after 4 weeks of transfer. +O = microbiota derived from old mouse; +Y = microbiota derived from young mouse. * = *p* < 0.05; ** = *p* < 0.01; *** = *p* < 0.001

Because it has been reported that basophils are regulated by gut microbiota [8], we determined differences in microbial genera (L6) between young and aged mice by 16S sequencing. *Alistipes*, *Bacteroidetes RC9 gut group*, *S24–7* family (L5), and *Oscillibacter* were significantly more abundant in aged mice compared with young mice, whereas *Lactobacillus* was significantly less abundant (Fig. 2a).

Next, we questioned whether the differences in basophils with aging are caused by gut microbiota. To this end, microbiota obtained from fecal samples of 4-month-old or 18-month-old mice were transferred to 3-month-old germ-free mice. After 1 week and after 4 weeks, we determined differences in microbial genera between germ-free recipients of young or old microbiota. After 1 week, the abundance of *Alistipes*, *Ruminococcus*, and *Akkermansia* was significantly decreased in recipients of old microbiota, compared with young microbiota, whereas *Lactobacillus* abundance tended to increase (Fig. 2b). After 4 weeks, the abundance of *Prevotellaceae unclassified* was significantly lower in recipients of old microbiota, compared with young microbiota, and the abundance of *Desulfovibrio* was significantly higher (Fig. 2c).

### No difference in basophil frequencies and phenotype after microbiota transfer of young and aged mice

**Fig. 3:**
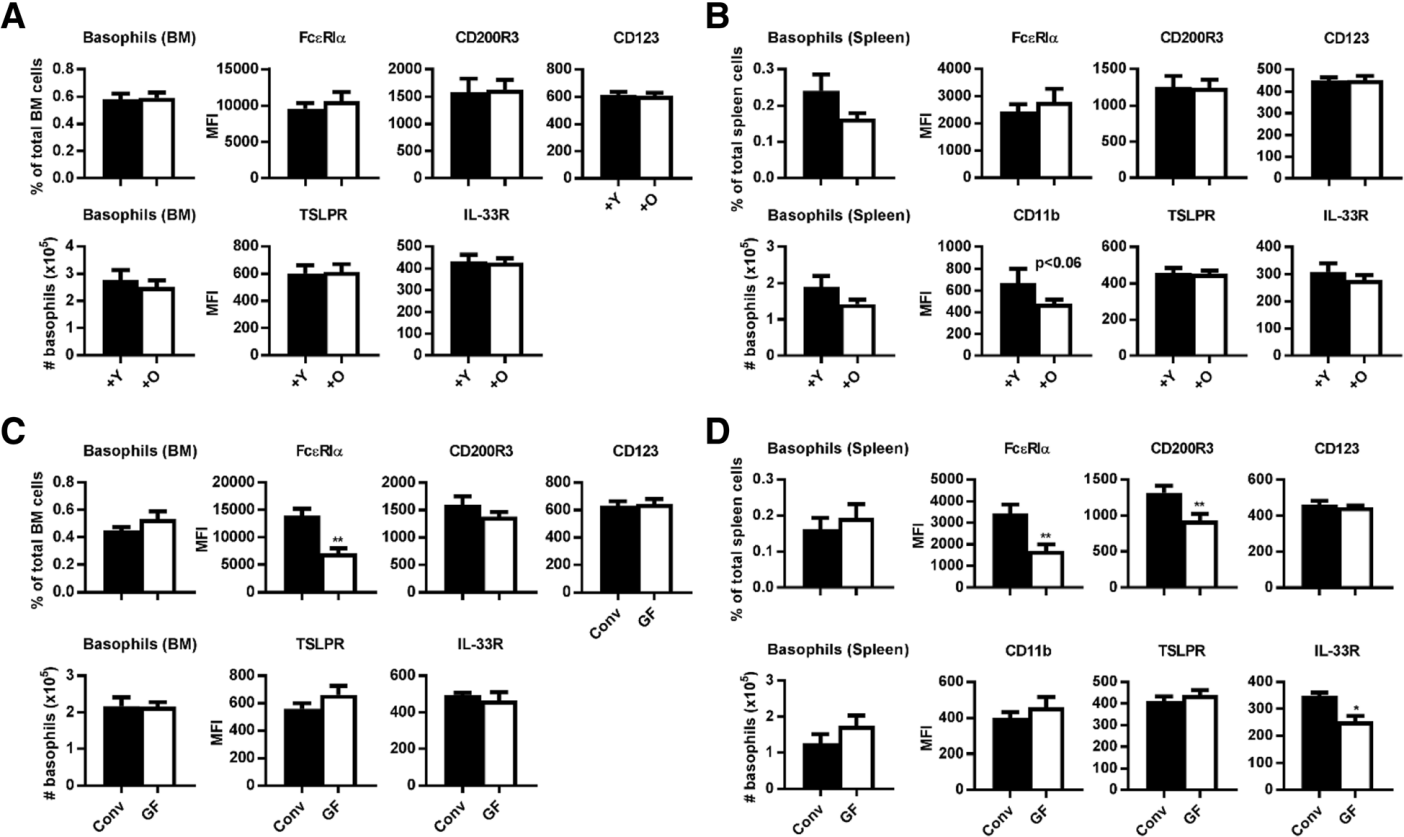
Effect of microbiota transfer of young and old mice to germ-free mice on basophil frequencies and phenotype in the bone marrow and the spleen. **a** Quantification of mean frequencies and absolute numbers of BM basophils or median fluorescence intensity (MFI) on BM basophils of FcεRIα, CD200R3, CD123, IL-33R, and TSLPR. **b** Quantification of mean frequencies of spleen basophils or MFI on spleen basophils of FcεRIα, CD200R3, CD123, TSLPR, IL-33R, and CD11b. **c** Quantification of mean frequencies and absolute numbers of BM basophils or median fluorescence intensity (MFI) on BM basophils of FcεRIα, CD200R3, CD123, IL-33R, and TSLPR. **d** Quantification of mean frequencies of spleen basophils or MFI on spleen basophils of FcεRIα, CD200R3, CD123, TSLPR, IL-33R, and CD11b. Data represent *n* = 10 mice per group (panel **a** and **b**) and *n* = 5 mice per group (panel **c** and **d**). BM = bone marrow; Conv = conventional; GF = germ-free; O = microbiota derived from old mouse; Y = microbiota derived from young mouse. * = *p* < 0.05; ** = *p* < 0.01

After microbiota transfers, we found no significant effects on frequencies, numbers, nor on phenotype of basophils (Fig. 3a, b). Both BM and spleen had similar basophil frequencies and numbers in the young or old microbiota recipients (Fig. 3a, b). In addition, no difference in FcεRIα, TSLPR, CD200R3, IL-33R, and CD123 was observed between young and aged recipient-mice. The only difference we observed was in splenic basophils that tended to express less CD11b in recipients of 18-month-old microbiota (*p* < 0.06; Fig. 3b). Because we did not observe changes in basophil distribution and phenotype after microbiota transfers, we checked whether the distribution and phenotype differed between conventional and germ-free mice. We found no differences between germ-free and conventional mice in terms of frequencies and absolute numbers of basophils. We confirmed that the absence of microbiota changed the phenotype of basophils. In particular, FcεRIα expression was more than 2-fold decreased on BM and splenic basophils from germ-free mice, in comparison with conventional mice (Fig. 3c, d). CD200R3 and IL-33R expression on splenic basophils was also decreased in germ-free mice (Fig. 3d).

### Association between basophil numbers or basophil phenotype, and abundance of microbial genera

**Fig. 4:**
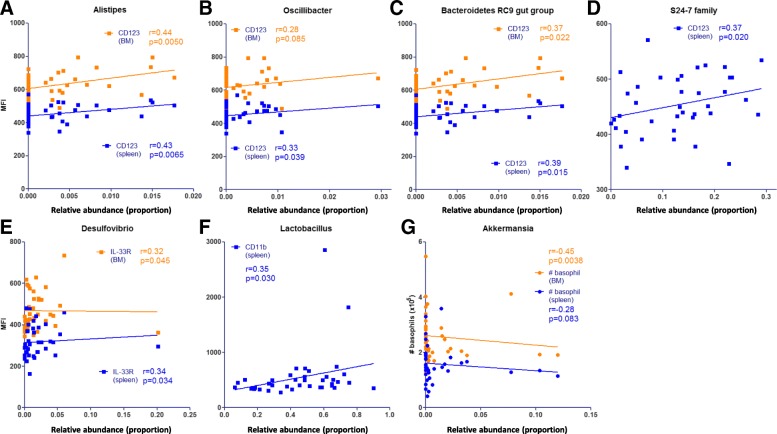
Correlation between basophil frequencies and phenotype, and abundance of microbial genera. **a**-**d** Spearman correlation between abundance of indicated microbial genera and CD123 expression in BM (orange) and spleen (blue). **e**-**f** Spearman correlation between abundance of indicated microbial genera and IL-33R or CD11b expression on BM and splenic basophils. **g** Spearman correlation between abundance of *Akkermansia* and absolute basophil numbers in BM and spleen. # = absolute cell counts; BM = bone marrow; MFI = median fluorescence intensity

Based on the microbial genera that were significantly different with aging or after microbiota transfers (Fig. 2), we subsequently investigated the association between gut microbiota and basophil frequencies and phenotype. We found that abundance of *Alistipes*, *Oscillibacter*, *Bacteroidetes RC9 gut group*, and *S24–7* family positively correlated with CD123 expression in BM and/or spleen (Fig. 4a, b, c, d). The abundance of *Desulfovibrio* positively correlated with IL-33R expression on basophils in BM and spleen (Fig. 4e). In addition, we found that abundance of *Lactobacillus* positively correlated with CD11b expression by splenic basophils (Fig. 4f). Finally, *Akkermansia* abundance negatively correlated with basophil numbers in BM and showed a similar tendency with splenic basophil numbers (Fig. 4g).

### In vitro function of bone marrow-derived basophils from old mice is impaired in part due to microbiota

Although frequency and the majority of the phenotypical markers were not influenced by the age of the microbiota, we wished to exclude that other differential functional parameters of the basophils were still intact. To this end we differentiated basophils in vitro from bone marrow and subsequently tested the functional response of purified basophils on several stimuli.

**Fig. 5:**
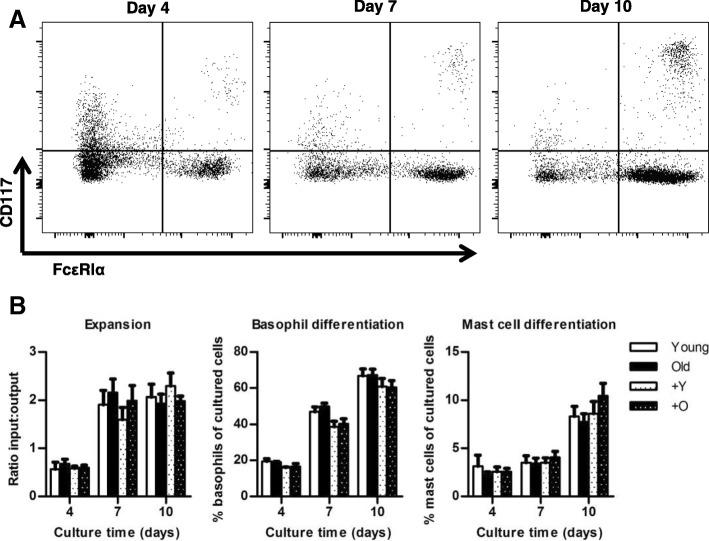
Effect of age and age-related microbiota on IL-3 BM cultures. **a** Representative gating of IL-3-driven BM culture, in which all live cells were gated for CD117 and FcεRIα. Basophils were defined as FcεRIα^+^CD117^−^ and mast cells as FcεRIα^+^CD117^+^. b Effect of age and microbiota on BM culture expansion and basophil and mast cell differentiation. Data represent *n* = 4 cultures per group for day 4 and *n* = 5 cultures per group for day 7 and 10 (with each culture derived from a different mouse). O = microbiota derived from old mouse; Y = microbiota derived from young mouse

Differentiation adequacy into FcεRIα^+^CD117^−^ basophils (and CD200R3^+^ basophils) or FcεRIα^+^CD117^+^ mast cells was determined by flow cytometry after 4, 7, and 10 days of culture (Fig. 5a). No differences in expansion of the whole culture, or differentiation were observed among the experimental groups (Table 1; Fig. 5b). About 98% of basophils were CD200R3^+^ after 10 days of culture (data not shown).

**Fig. 6:**
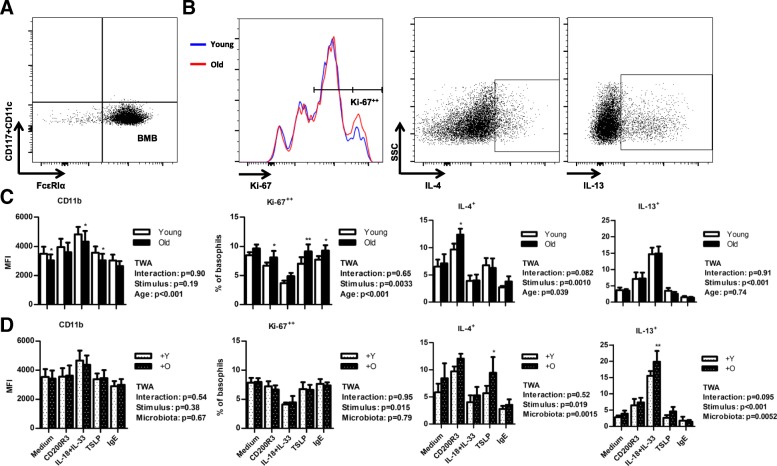
Effect of age and age-related microbiota on pure bone marrow-derived basophils. **a** Representative example of purified BMB, defined as FcεRIα^+^CD11c^−^CD117^−^. **b** Flow cytometric analysis of intracellular Ki-67, IL-4, and IL-13 staining of CD200R3-stimulated BMB. For Ki-67, a representative example of a young (blue) and an old mouse (red) is given. **c**-**d** Effect of stimulation with different stimuli on expression of (extracellular) CD11b and (intracellular) Ki-67, IL-4, and IL-13 by BMB from young and aged mice (panel **c**) and by BMB from germ-free recipients from young and aged mice (panel **d**). Every stimulation condition, including the medium control, contained 2 ng/mL IL-3. * = *p* < 0.05; ** = *p* < 0.01. Asterisks above bars indicate outcome of Bonferroni post hoc tests. The outcome of TWA is indicated below the legend. Data represent 5 cultures per group (with each culture derived from a different mouse). BMB = bone marrow-derived basophils; O = microbiota derived from old mouse; TWA = two-way ANOVA; Y = microbiota derived from young mouse

After 10 days of culturing BM cells with IL-3, we isolated the basophils (Fig. 6a). Purified BM-derived basophils (BMB) were overnight cultured under five different conditions: medium, IL-18 + IL-33, TSLP, IgE, or CD200R3. These conditions mimic different routes of activation of basophils [21]. The five different conditions resulted in distinct basophil phenotypes. IL-18 + IL-33 and CD200R3 were most potent in the induction of IL-4 and IL-13 by the basophils (Fig. 6b). For Ki-67, IL-4, and IL-13, but not CD11b expression, we observed a stimulus-dependent effect (Fig. 6c).

CD11b expression was decreased in BMB derived from 4-month-old mice compared with those from 18-month-old mice (*p* < 0.001; Fig. 6c). This was not microbiota-dependent, because CD11b was not altered in BMB derived from germ-free recipients of old microbiota compared with recipients of young microbiota (Fig. 6d). We originally planned to use Ki-67 as a measure of proliferation [34], but this was not applicable as most BMB were Ki-67^+^ (Fig. 6b). We therefore focused on a distinct cell population with high expression of Ki-67 (Ki-67^++^) as measure for proliferative activity. With aging, the frequency of Ki-67^++^ BMB consistently increased under all tested conditions (*p* < 0.001; Fig. 6c). The frequency of IL-4^+^ cells increased in old BMB (*p* < 0.05; Fig. 6c). This seemed to be influenced by microbiota, because similar differences were observed in BMB from recipients of old microbiota (*p* < 0.01; Fig. 6d). The IL-13^+^ frequency did not change with age (Fig. 6c), but did increase upon transfer of old versus young microbiota (*p* < 0.01; Fig. 6d). We compared the five culture conditions in aging, and after transfer of microbiota, but found the most pronounced effects in cultures stimulated with CD200R3, IL-18 + IL-33, and TSLP (Fig. 6c-d).

## Discussion

In this study, we found that basophil frequencies, numbers, and phenotype in the spleen change in mice during aging. Less effects on phenotype were found in the BM, although absolute numbers of basophils increased. This however should not be interpreted as a suggestion that no aging effects in the BM exist, as significant effects of age were found on the in vitro activation of basophils differentiated from precursors in the BM. Partly these in vitro effects were caused by the aging microbiota, as age-dependent changes in the activation of BM-derived basophil were also observed in young germ-free recipients of microbiota of 18-month-old mice. Fecal microbiota analysis showed that the microbiota composition significantly changed with age, and after microbiota transfers. Several microbial genera were correlated with basophil frequencies and phenotype.

Our report confirms age-related effects on basophils, showing for the first time that basophil phenotype changes. Intriguingly, CD123 expression by basophils from old mice consistently tended to increase. CD123 is crucial for IL-3 signaling and basophil hematopoiesis [29], and might explain the increased basophil numbers. IL-3 is, in higher amounts, also able to induce IL-4 production in basophils via the IL-3 receptor [35]. Aged basophils showed a tendency to lower expression of CD200R3, which inhibits FcεRIα-mediated activation of basophils [36]. CD200R3 also activates basophils to produce IL-4 and to degranulate [33]. Lower CD200R3 expression by basophils from aged mice (versus basophils from young mice) might indicate that aged basophils are less readily activated [33]. Together, these age-related changes might indicate an increased sensitivity to IL-3, and at the same time an altered threshold for activation. Thus, we were able to show differences in BM and spleen basophils with age.

Recently, we have shown a correlation between B cell precursors and abundance of specific microbial genera [37]. Similarly, in this study, we found an association between specific microbial genera and basophil frequencies and phenotype (both in BM and spleen). Most strikingly, the abundance of *Alistipes*, *Oscillibacter*, *Bacteroidetes RC9 gut group*, and *S24–7* family positively correlated with CD123 expression in BM and spleen. As indicated above, CD123 is crucial for basophil hematopoiesis and function. Transfer of specific microbiota into germ-free recipient mice would further support the association of microbial genera with basophil frequencies and phenotype we found in this study. Because basophils express TLR2 and TLR4 [13], it would be of high interest to determine expression of these receptors as well as responsiveness to their ligands in the context of aging.

To gain insight into the effect of aging on the precursors of basophils, we used IL-3-dependent BM cultures as a proxy (Fig. 5). First, we improved the method to generate basophils by at least 70-fold compared with a recent, detailed protocol [31]. Yoshimoto et al (2012) reported using femurs and tibias of ten 9- to 12-month-old Balb/c male mice. A conservative estimation of the starting number of BM cells in their cultures is 4 × 10^8^, which resulted in 20-40 × 10^6^ cultured cells (culture efficiency ≤10%). After purification, 1-4 × 10^6^ basophils were collected (purification efficiency ≤10%). Under the best conditions, the mentioned protocol ends with a 1% yield. In our hands, the culture efficiency of the improved BMB generation protocol was higher than previously reported, with each 10^6^ BM cells generating on average 2 × 10^6^ cultured cells. Taking into account the withdrawal of cells for direct assessment three times during the culture, our culture efficiency was a bit higher than 200%. Our purification method, which includes dendritic cell removal, resulted in higher numbers of pure basophils: we isolated on average 6.9 × 10^6^ pure basophils per 20 × 10^6^ cultured cells (35% purification efficiency). Regardless different origins of BM (Table 1), our protocol ends with an average yield of 70%. The vast difference between the yields are most likely explained by the cell density at the start of the culture. Other differences that might cause improved yield are mouse strain, fresh versus frozen BM, and the purification method. Thus, using our robust method, we were able to assess basophil function by using a few million BM cells as input. It is important to underline the importance of excluding the adherent cells during the culture and the targeted depletion of CD11c^+^ dendritic cells during the isolation of BMB. This enables to specifically look at BMB responses, without bystander effects of stromal cells or dendritic cells.

We identified additional differences between young and aged BMB (Fig. 6). CD11b expression was decreased, whereas IL-4^+^ (but not IL-13^+^) frequencies were increased upon activation in BMB from aged mice. IL-4^+^ basophil frequencies were particularly increased after CD200R3 stimulation, in line with previous studies [33]. BMB derived from germ-free recipients receiving microbiota of aged mice (versus microbiota of young mice) also showed increased IL-4^+^ basophil frequencies. Thus, we found that microbiota from aged mice influence basophil precursors and subsequent in vitro activation.

The functional implications of these findings remain to be elucidated. It is conceivable that basophils may differ in their functional response in vivo, because Hill et al (2012) showed that antibiotics under steady state conditions in vivo did not alter basophil frequencies in lymph nodes. Basophil frequencies, however, were increased after papain treatment in antibiotic-treated mice (compared with control mice) [8]. Allergic challenges or helminth infections in young versus aged mice would give insight in the functional consequences in vivo of the observed changes between young and aged basophils, and after microbiota transfers of young and aged mice.

Our study has a number of limitations: 1) Due to the relatively small populations of basophils and the required numbers of aged and germ-free mice, we were not able to sort basophils directly from spleen or bone marrow to evaluate in vitro basophil function. 2) We could not study alterations in in vivo production of e.g. IL-4 by basophils with aging, as could be done by using aged or germ-free IL-4-eGFP reporter mice. 3) We used total aging-associated microbiota, rather than selected microbial strains that were altered upon aging and correlated with basophil phenotype or numbers.

## Conclusions

Our study shows that aged mice display increased basophil numbers and altered phenotype, which seems independent of aging-associated microbiota. In vitro activation of BM-derived basophils is impaired with aging, which in part is explained by aging-associated microbiota. Further functional in vivo studies are warranted to investigate the consequences of our findings for Th2-mediated immune responses in aging.

## Methods

### Mice

Young and old wild-type C57Bl/6 mice were purchased from Harlan (Horst, The Netherlands). Germ-free C57Bl/6 mice were generated at the Central Animal Laboratory of the Radboud University Medical Center (Nijmegen, The Netherlands). Mice were kept in individually ventilated cages or sterile incubators, and were specific pathogen free (SPF). All mice had free access to feed (ssniff, rat/mouse maintenance V153X R/M-H) and water. All groups consisted of *n* = 10 mice, unless otherwise mentioned. We have used mice as an animal model, because most tools are available for this animal model. We have used 19–20-months-old mice as aged, because many age-related changes have been reported to occur already at that age, and because tumor incidence increases after 20 months [38, 39].

### Microbiota transfers

Feces from 4-month-old and 18-month-old female mice were freshly collected. Part of the feces was stored for microbial analysis, the remaining part was mixed with PBS. Three-month-old germ-free mice were administered 200 μL of 100 mg/mL fecal solution by intragastric gavage (20 mg/mouse). These mice were then housed in IVC for another month.

### Organ collection and cell suspensions

At 4–5 months or 19–20 months of age, mice were anesthetized with isoflurane, bled, and sacrificed by cervical dislocation. Serum was collected by spinning the clotted blood, and was stored at − 80 °C until further analysis. Mice were inspected for visible tumors, which lead to the exclusion of one aged mice. Femurs and spleen of each mouse were isolated. Single-cell suspensions of BM were obtained by flushing the femurs, whereas the spleen was cut in pieces. Cells were then passed through a cell strainer. Part of the BM cells were frozen for later use in vitro.

### Flow cytometry

Flow cytometry was performed using standard procedures. After staining for surface markers, cells were incubated with live/dead eFluor506 or eFluor520 stain (Ebioscience). Cells were then fixed using the FoxP3/Transcription Factor Staining Buffer kit (Ebioscience), with the exception of the Golgi-Stop-treated cells. They were processed using the Intracellular Fixation and Permeabilization kit (Ebioscience) to preserve intracellular cytokines. Used antibodies are listed in Table 2. Flow cytometric measurements were acquired by a FACSCanto II flow cytometry (BD Biosciences, Erembodegem, Belgium). FlowJo software vX.07 (Tree Star, San Carlos, USA) was used for data analysis.

**Table 1:** Average input, output, yield, and purity of basophils from IL-3 BMB cultures

Group	Input BM cells ×10^6^	Output cultured cells ×10^6^	Yield pure basophils ×10^6^	Purity %
Young	5.6 (0.4)	11.6 (1.9)	4.5 (1.1)	97 (1)
Old	6.0 (0.0)	11.6 (1.1)	3.2 (1.3)	97 (1)
+Y	6.0 (0.0)	13.7 (1.6)	3.7 (1.2)	97 (1)
+O	5.6 (0.4)	11.0 (0.7)	3.4 (1.0)	95 (2)

Data represent 5 cultures per group (with each culture derived from a different mouse). Standard error of the mean between brackets

*BM* bone marrow, *BMB* bone marrow-derived basophils, *+O*, microbiota derived from old mouse, *+Y* microbiota derived from young mouse

**Table 2:** Used antibodies for flow cytometry and purification

Target	Format	Clone	Company
CD3e	FITC	145-2C11	BD
CD4	FITC	H129.19	BD
CD8a	FITC	53–6.7	BD
CD11b	BV421/FITC	M1/70	BD
CD11c	Biotin/FITC	HL3	BD
CD16/32	FITC/Purified	2.4G2	BD
CD19	FITC	1D3	Ebioscience
CD45R/B220	FITC	RA3-6B2	BD
CD62L	APC-Cy7	MEL-14	BD
CD117	Biotin BV421 BV510	2B8 2B8 ACK2	BD BioLegend BioLegend
CD123	Biotin PE	5B11 5B11	BD Ebioscience
CD200R3	APC	Ba13	BioLegend
FcεRIα	Biotin/PE-Cy7	MAR-1	Ebioscience
IL-4	APC	11B11	Ebioscience
IL-13	PE-Cy7	eBio13A	Ebioscience
IL-33R/ST2	PerCP-eFluor710	RMST2–2	Ebioscience
Ki-67	FITC	SolA15	Ebioscience
Ly6C/Ly6G (Gr1)	FITC	RB6-8C5	BD
NK1.1	FITC	PK136	Ebioscience
TER-119	FITC	TER-119	BD
TSLPR	PE		R&D
Streptavidin	APC-eFluor780		Ebioscience

### 16S sequencing

At sacrifice of all mice, fecal pellets from colon were sampled, snap frozen in liquid nitrogen, and stored at − 80 °C. These samples were used for 16S rRNA gene analysis for microbiota profiling, as further described in Fransen et al. 2017 [40]. Microbial genus (L6) data were used throughout this manuscript, unless otherwise indicated.

### Basophil generation and stimulation in vitro

BM cells were thawed, checked for viability by trypan blue, and counted. BM cells were cultured, using an optimized method that was adapted from a previously published protocol [31]. About 3.3 × 10^5^ viable BM cells per mL culture medium were plated in 6-wells plates. Culture medium consisted of RPMI-1640 medium (Gibco, Breda, The Netherlands), 10% fetal calf serum (Gibco), 100 μg/mL Normocin (Invivogen, San Diego, USA), 2 ng/mL rmIL-3 (Sanquin, Amsterdam, The Netherlands), and 50 μM β-mercaptoethanol (Sigma-Aldrich, Zwijndrecht, The Netherlands). Cells were cultured for 10 days. Every 3–4 days, non-adherent cells were collected, counted, and re-plated. About 10^5^ cells were used for flow cytometry to measure proliferation and differentiation in the cultures (see Table 1 for antibodies). Expansion of each culture was calculated by dividing the cell count by the input. After 10 days, cells were incubated with purified anti-CD16/32 and subsequently with biotinylated CD11c and CD117 (all BD Biosciences, San Jose, USA). Cells were then incubated with streptavidin-coated IMag beads (BD) and processed with the IMagnet (BD). The negative fraction was incubated with biotinylated FcεRIα and subsequently with streptavidin-coated IMag beads and processed with the IMagnet. The positive fraction (containing CD11c^−^CD117^−^FcεRIα^+^ cells) were defined as BM-derived basophils, and purity typically exceeded 95% (average > 96%). Pure BMB were resuspended to 5 × 10^5^/mL and stimulated for 15 h with culture medium (including IL-3) alone, 1 μg/mL rmTSLP (Ebioscience, San Diego, USA), 5 μg/mL CD200R3 (BioLegend, San Diego, USA), 10 μg/mL IgE (Abcam, Cambridge, USA) or a combination of 50 ng/mL rmIL-18 (MBL International, Watertown, USA) and 100 ng/mL rmIL-33 (Sanquin). For intracellular cytokine staining, cells were stimulated for 11 h, and Golgi-Stop (BD) was added for an additional 4 h.

### Statistical analysis

All statistical analyses were performed in Prism 5.0 (GraphPad Software, San Diego, USA). For comparing two experimental conditions, unpaired Student’s *t* test was applied (with Welch’s correction if unequal variances were observed). Mann-Whitney *t* test was applied if no normal distribution was found with D’Agostino & Pearson omnibus normality test. Median fluorescence intensities were tested by paired Student’s *t* test or Wilcoxon signed rank test (in absence of normal distribution), because all experimental groups were equally distributed at any day for acquisition. Correlations were determined by Spearman’s rank correlation. If testing the effect of two variables and their interaction (e.g. culture time and age), two-way ANOVA (TWA) was applied, with Bonferroni post hoc tests (normality verified by Kolmogorov-Smirnov normality test). Values of *p* < 0.05 were considered to be statistically significant, and values between *p* > 0.05 and *p* < 0.10 were considered to be a trend. Significant differences are indicated by asterisks: * = *p* < 0.05; ** = *p* < 0.01; *** = *p* < 0.001.

## Abbreviations

BAFF: B cell activating factor
BM: bone marrow
BMB: bone marrow-derived basophils
CCL: C-C motif chemokine ligand
GF: germ-free
GM-CSF: granulocyte-macrophage colony stimulating factor
Ig: immunoglobulin
IL: interleukin
Lin: lineage
LPS: lipopolysaccharide
SPF: specific pathogen-free
Th: T helper
TLR: Toll-like receptor
TSLP: thymic stromal lymphopoietin
TWA: two-way ANOVA
